# Evaluation of tuberculosis surveillance system in a municipality in Ghana during the COVID-19 pandemic: a cross-sectional study

**DOI:** 10.11604/pamj.2024.48.170.40303

**Published:** 2024-08-12

**Authors:** Alex Ansah Owusu, Priscilla Anima Poku, Andrews Ayim, Jeffrey Kojo Arhin, Richard Nii Armah, Alfred Edwin Yawson

**Affiliations:** 1 Department of Public Health, Pantang Hospital, Accra, Ghana,; 2 Municipal Health Directorate, La Nkwantanang Madina Municipality, Accra, Ghana,; 3 Policy, Planning, Monitoring and Evaluation Division, Ghana Health Service, Accra, Ghana,; 4 Municipal Health Directorate, Adentan Municipality, Accra, Ghana,; 5 Department of Public Health, St. Andrew Catholic Hospital, Shai-Osudoku, Ghana,; 6 University of Ghana Medical School, Accra, Ghana

**Keywords:** Surveillance, surveillance system evaluation, tuberculosis

## Abstract

**Introduction:**

public health surveillance is the ongoing systematic identification, collection, collation, analysis, and interpretation of disease occurrence and public health event data, to take timely and robust action, such as disseminating the resulting information to the relevant people, for effective and appropriate action. Tuberculosis (TB) is an infectious disease caused by the micro-organism Mycobacterium tuberculosis. The main objective of this study was to describe the operation and performance of the TB surveillance system in a municipality in Ghana during the COVID-19 pandemic.

**Methods:**

this was a cross-sectional study, which employed qualitative and quantitative data collection methods. The process was guided by the Updated Centres for Disease Control and Prevention (CDC) Surveillance System Evaluation Guidelines. The study was conducted in the La Nkwantanang Madina Municipality (LaNMMA).

**Results:**

the system was found to be useful for planning, monitoring, and evaluation of TB control activities as well as the development of priorities for TB control programmes. The system was found to be simple and flexible with good data quality. However, stability, sensitivity (44.2%), predictive value positive (8.7%) and acceptability were all found to be poor. Even though the downstream flow of information was found to be excellent, the upstream flow of information was found to be poor.

**Conclusion:**

it was concluded that the system was performing poorly. The system was not achieving most of the objectives for which it was set up. The Ghana Health Service should take measures to strengthen and improve the LaNMMA TB surveillance system in the post-pandemic era.

## Introduction

Public health surveillance is the continuing, systematic identification, collection, collation, analysis, and interpretation of data on disease occurrence and public health events to prompt action that is both effective and appropriate [1]. Moreover, surveillance is crucial for public health practice development, implementation, monitoring, and evaluation.

Tuberculosis is an infectious disease caused by the micro-organism *Mycobacterium tuberculosis* (MTB) [2]. The micro-organisms usually enter the body when inhaled into the lungs [3]. The human race has been afflicted by tuberculosis (TB) for more than 4,000 years [4]. TB is one of the top 10 global killers and the leading infectious-agent killer, surpassing HIV/AIDS as the leading cause of death [5]. It causes millions of deaths worldwide each year [6].

In 2019, an estimated 10 million individuals developed TB, and 1.4 million succumbed. In the same year, 87% of the people who fell sick with TB were in 30 high TB-burden nations [5]. At the national level, the rate of cases varies from fewer than 5 to more than 500 per 100,000 people each year. According to a 2013 TB prevalence survey, Ghana´s estimated TB prevalence was 290 per 100,000 population. This indicated that the burden of TB in Ghana was 4 times higher relative to the World Health Organization (WHO) estimates of 71 per 100,000 population for the same year [7].

During the 2019 TB situation in Ghana, 44,000 persons contracted the disease, including 6,300 children. Moreover, 29,309 TB patients were thought to have been lost to follow-up. Five thousand four hundred and seventy-seven of those were children. Around 1,200 persons reportedly developed drug-resistant TB. Nine hundred and eighty-three drug-resistant TB patients were discovered to have been lost to follow-up. HIV infection was present in 9,200 TB patients, according to estimates. Fifteen thousand persons passed away from TB-related causes [8].

The tuberculosis surveillance system forms a part of the Integrated Disease Surveillance and Response (IDSR) system which works within the decentralized government health service delivery system in Ghana. The goal of the TB surveillance system in LANMMA is the early diagnosis and treatment of all TB patients in the municipality as well as providing data to serve as a basis for policymakers' decisions about the control and eradication of TB in the nation.

As of the time of this evaluation, the objectives of the LANMMA TB surveillance were: 1) to early screen, detect and enrol in treatment all forms of notified (new cases) from 15,606 in 2013 to 37,302 by 2020, while increasing the proportion of bacteriologically confirmed pulmonary TB from 51% in 2013 to 60% by 2020; 2) to early detect and enrol into treatment at least 85% of confirmed MDR-TB cases among new and previously treated cases by 2020; 3) to attain higher treatment success for all forms of TB from 84% in 2012 to at least 91% by 2020 through improved quality clinical care and community TB care; 4) to reduce death rates of TB/HIV co-infected cases from 20% in 2012 to 10% by 2020 and uptake of antiretroviral therapy (ART) coverage among co-infected from 37% in 2013% to 90% by 2020; 5) to improve programme management; coordination monitoring and evaluation and operations research to support treatment and screening strategies for TB/HIV [9].

Information from the LANMMA TB surveillance system will be used to: 1) promote research that will help improve the existing system; 2) inform the procuring and distribution of the resources used in running the system; 3) monitor and evaluate the TB surveillance activities.

The objectives for the evaluation of the existing surveillance system were to describe the operation, and performance and identify any gaps in the TB surveillance system in the selected municipality in Ghana.

## Methods

**Study design:** a descriptive cross-sectional study using both qualitative and quantitative approaches was conducted. The process was guided by the updated Centre for Disease Control and Prevention (CDC) Surveillance System Evaluation Guidelines.

**Study setting:** the study was conducted in the La Nkwantanang Madina Municipality (LANMMA), one of the 29 municipalities in the Greater Accra Region of Ghana. The Greater Accra Region was purposively selected because it had recorded the highest number of COVID-19 cases in Ghana at the time of the study. LANMMA was purposively selected because this municipality has urban, peri-urban and rural settlements. This municipality is also densely populated. The municipality has five sub-municipalities for the administration of health care services. There are 38 demarcated Community-Based Health Planning and Services (CHPS) zones. The projected population for the municipality for the year 2020 was 137,706. The period evaluated spanned from January 2018 to December 2020.

**Study participants and sampling:** the study participants included facility administrators, doctors, general nurses, public health nurses (PHN), community health nurses (CHN), community health officers (CHO), enrolled nurses, biomedical scientists, pharmacists, a radiographer, health information officers, a TB patient, courier service rider and team at municipal health directorate. Table 1 gives details of the stakeholders that were interviewed. Purposive sampling was used to select the participants who could provide the relevant information for the study, based on their unique characteristics, experiences and knowledge. Individuals who were identified as key informants in the TB surveillance system were interviewed with the aid of an interview guide.

**Table 1:** stakeholders that were interviewed

Stakeholders	No. interviewed
Doctors	4
Physician assistants	2
Facility heads	3
Facility TB coordinators	5
Task shifting officers	2
TB patients	1
Pharmacist (government hospital)	1
Pharmacist (private pharmacy)	1
DDNS	2
Facility health information officer	1
Courier service	1
CBSV	1
Radiographer	1
Biomedical scientist	4
Facility health promotion officer	1
CHO	2
Municipal TB coordinator	1
Municipal disease control officer	1
Municipal DDNS	1
Municipal public health nurse	1
Municipal CHPS coordinator	1
Municipal accountant	1
Regional technical officer for TB (NTP staff)	1
Regional TB coordinator	1
Total	40

TB: tuberculosis; DDNS: deputy director of nursing services; CBSV: community-based surveillance volunteer; CHO: community health officer; CHPS: community-based health planning and services; NTP: National Tuberculosis Control Programme

**Stakeholders:** the stakeholders identified as relevant to the TB surveillance in LANMMA at the municipal level included the municipal director of health services (MDHS), the municipal TB coordinator, the municipal disease control officer, the municipal public health nurse (MPHN), the municipal pharmacist, the municipal health promotion officer, the municipal deputy director of nursing services (DDNS), the municipal accountant, the municipal CHPS coordinator and the municipal human resource manager (HR).

The community and facility-level stakeholders included doctors, physician assistants, pharmacists, biomedical scientists, radiographers and radiologists. Also identified were the institutional tuberculosis coordinators, health information officers at the facility level, disease control officers, community health nurses (CHNs), community health officers (CHOs) and community-based surveillance volunteers (CBSVs). Community pharmacists (drug store operators) and TB patients were also identified as stakeholders. The TB surveillance system stakeholders identified at the regional level were the regional TB coordinator, the deputy director of public health and the regional director of health.

Stakeholders identified at the national and international level included: Ghana Health Service, the Christian Health Association of Ghana (CHAG), the National Health Insurance Authority (NHIA), the National Tuberculosis Control Programme (NTP), the National AIDS Control Programme (NACP), the Ministry of Health (MOH), World Health Organization (WHO), US Agency for International Development (USAID), Global Fund to Fight AIDS, Tuberculosis and Malaria (Global Fund).

**Data collection:** data for the evaluation were collected by records review using data compilation forms and key informant interviews using an interview guide. The interview guide asked questions on case definitions, target population, objectives of the surveillance system, flow of communication in the surveillance system, data collection and reporting, measures in place to ensure patient privacy and nature and frequency of data analysis. TB records in the five directly observed treatment, short-course (DOTS) facilities as well as the LANMMA health directorate were reviewed. Some of the specific documents that were reviewed are cough registers, laboratory and X-ray request forms, laboratory registers, treatment cards, institutional TB registers, the municipal TB register, case-based forms, monthly TB reports and the District Health Information Management System 2 (DHIMS II) database. These sources were reviewed for the number of TB cases suspected, number of cases confirmed, case detection rate, cure rate, treatment success rate, default rate and fatality rate. The level of completeness of specific variables in the institutional TB register for the various TB treatment centres was also observed. Data collection was from 4^th^ January 2021 to 31^st^ March 2021.

**Data management and analysis:** the recordings were transcribed verbatim and typed into Microsoft Word. System attributes that were observed were documented. Data from the data compilation forms were entered into Microsoft Excel and exported to Epi-Info version 7.2.4 for analysis. Data were presented as tables. The findings from the evaluation were reviewed by the municipal TB coordinator and director of health.

**Ethical considerations:** approval to carry out this evaluation was obtained from the LANMMA municipal director of health services. Letters were sent to all the facilities that were involved in the evaluation to inform them of the evaluation and its relevance.

**Informed consent:** informed consent was obtained from participants.

All identifiers that would allow for the linking of data to individuals were removed from the data. Electronic versions of the data were saved under a password and hard copies were stored in a locked cabinet that could be accessed by only the principal investigator.

## Results

**Description of the system:** the TB surveillance process in LANMMA begins at the community level with an active search for cough cases at the community level by Community Health Nurses (CHNs) and Community Health Officers (CHOs). They do this by sensitizing community members through TB health education at community information centres, schools, churches, mosques, home visits and community durbars (most of these people man CHPS zones and hence carry out TB education as they do their other routine tasks). Health promotion officers and DOTS nurses in DOTS centres also occasionally engage in TB health education at information centers and organized durbars. Suspected TB cases in the community report to the nearest health facility (either public or private) where they are referred to one of the five TB treatment centres in LANMMA for confirmation and treatment.

At the treatment centres, suspected TB cases are directed straight to the facility DOTS corners. These clients do not join the regular OPD queues. Three of the 5 DOTS facilities visited were observed to have task-shifting officers who sat at OPDs and looked out for OPD attendants who were coughing. They screen such patients with the TB screening tool, those identified as suspected TB cases were entered into the presumed TB register and referred to the DOTS corner.

All confirmed HIV/AIDS clients as well as contacts of known TB patients were also referred to the DOTS corner. At all the 5 DOTS centres in the municipality, sputum samples are taken from suspected cases and sent to the only laboratory with a Gene Xpert MTB/RIF facility (Madina Polyclinic-Kekele) which is the TB hub centre in the municipality. The samples are sent through a sputum transport system (a 2019 initiative by NTP to improve the efficiency of the system). The courier service responsible for the sputum transport is expected to visit the DOTS centres for sample collection on Wednesdays and Fridays. Sputum samples are stored at the labs of the spokes centres until they are picked up by the courier services and transported to the hub centre. Lab results are initially shared via WhatsApp with facility TB coordinators followed by hard copies.

When the lab results get to the facility TB coordinators, they invite the positive cases via phone calls. Cases are encouraged to visit with a close relative or a trusted friend who usually becomes the treatment supporter. Confirmed cases are registered and put on treatment. Institutional TB coordinators at the treatment centres compile data on all cases and report to the municipal TB coordinator on a monthly and quarterly basis. At the level of the municipal health directorate, the municipal TB coordinator compiles data from all the treatment centres, updates the information in DHIMS 2 monthly, and submits compiled data to the regional TB coordinator at the regional health directorate quarterly. At the regional level, quarterly reports from all the districts and municipalities in the region are collated and submitted to the national TB programme manager.

Information dissemination occurs bidirectionally at the various levels of the surveillance system, from the national to the community level. At the national level, quarterly review meetings are organized for regional TB coordinators, deputy directors (public health) and regional directors of health services from around the country. At the regional level, review meetings are organized quarterly for district directors of health services, district TB coordinators and institutional TB coordinators. At the municipal level information is disseminated through quarterly review meetings attended by facility in-charges, laboratory staff, community health nurses, and other stakeholders to discuss TB and provide feedback on issues regarding TB management in the municipality.

**Figure 1:**
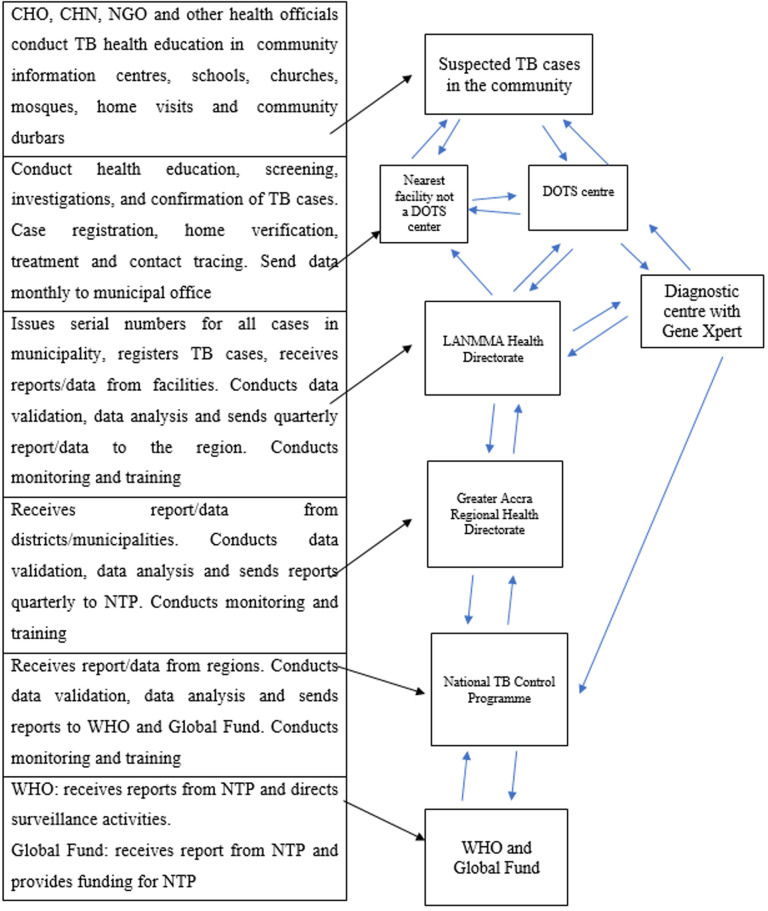
information flow in the La Nkwantanang Madina Municipality (LANMMA) TB surveillance system

Apart from these pre-arranged meetings, timely communication occurs at all levels in the system via the use of WhatsApp-created platforms. These platforms allow for the exchange of all forms of information such as pictures, word documents, PDF documents, MS Excel documents, videos, audio, text messages and even locations. Meetings are sometimes held via Zoom or Microsoft Teams. Figure 1 shows the information flow in the LANMMA TB surveillance system.

**Gaps identified during the evaluation period:** it was found that lab results were usually delayed to about 2 weeks or never returned. This current evaluation revealed that the results of 33.6% of samples sent to the hub lab in 2020 were not received [10]. This percentage does not include the sputum samples discarded at the DOTS centres because the courier services did not pick them up for transportation to the lab (samples not tested after two weeks of collection become non-viable and are therefore discarded). Due to the inconsistencies in the sputum transport system and operations of the hub lab, various adaptations were observed at the different DOTS centres to keep the system running.

Based on the prescriber who saw the suspected TB cases at one facility, some were put on TB treatment using chest radiograph (CXR) findings (guided by artificial intelligence, CAD4TB). Most of the time, the facility TB coordinator had to call a contact at the hub lab to follow-up on samples and hopefully send pictures of the results via WhatsApp when they were ready. The TB coordinator would then send for the hard copy of the results usually at her own cost. At another facility, the facility TB coordinator explained that he sent collected sputum samples to a neighbouring district´s hud lad where he usually got the results in about 2 hours. He did this at his own cost.

At a third facility, several sputum samples got discarded because the courier service didn´t come for samples hence suspected cases are sometimes started on treatment based on clinicians´ clinical judgment.

A member of the TB team at the hub centre explained that she usually went to the head of the lab, who ensured she got her results, sometimes on the same day. However, she added that results for samples taken for treatment follow-up in months 2, 5 and 6 were usually delayed, so she was forced to fill those spaces in the register with pencil for the sake of monitoring and evaluation visits. Symptom-based forms that were supposed to be available and used at all Out Patient Departments (OPDs) in facilities were only found in TB and HIV clinics. In facilities with task-shifting officers, these forms were also found at the medical OPDs. The TB coordinators had stopped placing the forms at the other units because health workers not directly part of the TB team were not interested in filling those forms so with time the practice stopped. Some TB clients who had been transferred to LANMMA (due to proximity) for continuation treatment were found to report late. Some only reported after symptoms had worsened.

**Evaluation of data analysis and use:** this study found that only one of the DOTS facilities carried out a computer-based analysis of data and used findings to inform decisions at the facility level. Various indicators and variables were analysed for trends. This facility also did a comparative analysis of different months, quarters and years. The TB coordinator in another facility said she analyzes the data occasionally by visual inspection. At the municipal, regional and national levels, data are analyzed monthly, quarterly, and yearly. Various indicators and variables such as the number of TB patients tested for HIV, number of samples taken per month, case notification rates, cure rates, number of pediatric TB cases diagnosed and number of multi-drug resistant TB (MDR-TB) diagnosed were analyzed for trends. In addition, comparative analysis between different months, quarters and years was done at these levels. The findings were used to evaluate how the system was performing. Analysed data were also used to assess interventions introduced in the programme.

**Cost of operations:** both human and financial resources were needed to run the TB surveillance system. TB surveillance is integrated with the general health care system of Ghana. The TB surveillance system depends mainly on the facilities and staff in the public healthcare system for its surveillance activities. The main funding agency for the system is the Global Fund. It funds the bulk of the expenditure on administration, training, monitoring and evaluation, logistics and feeding of clients. The government of Ghana also contributes to the surveillance system by paying the salaries of most of the workers. Occasionally some TB training and active community-based case searches were funded by non-governmental organisations (NGOs).

This study found that the TB surveillance system shared its workforce with other surveillance systems running in the municipality. However, apart from the task-shifting officers who worked solely for the TB surveillance system, all other staff dedicated their time in different proportions to the execution of the operations of the TB surveillance system. The estimated cost of running the surveillance system on a yearly basis was globally harmonized system (GHS) 131,768.84, consisting of personnel cost of GHS 48,915, direct cash funding of GHS43,984 and material cost of GHS 34,975. Using the number of cases confirmed in 2020, the cost of detecting a single case of TB was calculated as 131,768.84÷75= GHS 1,756.92.

### System performance

**System usefulness:** the TB surveillance system was found to be fairly useful. It detected cases for accurate diagnosis, treatment and contact tracing. The system also provides estimates of the magnitude of morbidity and mortality related to TB.

Data from the TB surveillance system was mainly used for planning, monitoring and evaluation of TB control activities and the development of priorities for TB control programmes. Information from the TB surveillance system helps with the allocative efficiency of scarce resources. The number of cases identified within a particular period helps to determine the quantities of materials and logistics required to run the surveillance system. The number of surveillance forms, patient treatment cards, TB medications, laboratory reagents and other supplies requested from the regional health directorate is informed by the number of notified cases. Table 2 shows some relevant indicators obtained from the TB surveillance system.

**Table 2:** three-year trend of some key performance indicators of the TB surveillance system in La Nkwantanang Madina Municipality (LANMMA): 2018-2020

Indices	National target	2018	2019	2020
No of cases detected		101	94	75
Case detection target		200	204	209
Case detection rate	60%	50.5%	46.1%	35.9%
TB treatment success rate	90%	76.9%	75%	70%
TB cure rate	100%	45%	65%	-
Fatality rate	5.0%	6.2%	10%	0%
Default rate (lost to follow-up)	0.0%	3.6%	4%	-
Treatment failure	<1%	0.9%	0.3%	-

**System attributes:** the attributes of the LANMMA TB surveillance system that were evaluated were: 1) simplicity, which refers to both its structure and ease of operation; 2) flexibility refers to its capacity to adjust to changing operational requirements or information needs with little additional time, staff, or budgetary resources; 3) stability which refers to the ability of the system to function reliably and consistently over time; 4) sensitivity, answers the question, of those who truly have the disease, what proportion did the surveillance system detect; 5) predictive value positive which refers to the proportion of reported cases that were positive for TB; 6) data quality which reflects the completeness and validity of the data recorded in the public health surveillance system; 7) timeliness which is the speed between steps in the surveillance system; 8) acceptability which refers to the willingness of persons and organizations to participate in the surveillance system; 9) representativeness, refers to the system´s ability to describe the occurrence of TB over time and its distribution in the population by place and person.

A detailed description of the attributes of the LANMMA TB surveillance system is shown in Table 3.

**Table 3:** description of the La Nkwantanang Madina Municipality (LANMMA) TB surveillance system attributes based on data from 2018-2020

Attributes	Findings	Remarks
Simplicity: refers to both its structure and ease of operation	Simple case definition that is widely known by users of the system; the tools for registration and reporting are easy to use; it takes an average of 5 minutes to fill the TB symptom-based screening tool	Very simple
Flexibility	The system adapted well to several changes such as the introduction of a sputum transport system, and the use of Gene Xpert MTB/RIF for diagnosis instead of microscopy; the system also runs very well with the HIV surveillance system with little or no additional resources	Flexible
Data quality	Almost all required fields in the institutional and municipal TB registers were validly completed. This was the same for the lab TB registers	Good data quality
Sensitivity	The number of TB cases detected x 100, in 2018 was 50.5%, in 2019 was 46.1% and in 2020 was 35.9%; the total number of TB cases expected gave a mean of 44.2% for the evaluation period	Low sensitivity
Predictive value positive	The number of suspected cases of TB confirmed as positive (270) x100 = 8.7%; the total number of suspected cases of TB (3,087)	Poor
Timeliness	TB clients stayed in the community for about 2 weeks to one month before reporting to health facilities; from contact with a health facility to diagnosis took about 2 weeks - 4 weeks. There were instances where the diagnosis was never made; from diagnosis to notification of LANMMA Health directorate average took a month; the downward flow of information was seamlessly done by sharing the information on created WhatsApp platforms that had the contacts of the stakeholders that needed the particular information	Upward flow poor, downward flow excellent
Acceptability	Poor timeliness of the upward flow of information; most health workers in LANMMA who were not directly part of the DOTS team were not interested in the activities of the TB surveillance system	Poor
Representativeness	The cases were not evenly distributed over the three years under review: the proportion of males registered as TB patients was higher than females; of the 270 cases detected only one was a pediatric case constituting only 0.4% of detected cases; most of the confirmed cases were from Madina	Not representative
Stability	The sputum transport system was found to be very inconsistent and unreliable; a significant proportion of samples are missing at the lab (33.6% of 2020 samples); some activities are paid for by staff with their personal money. Hence whenever staff chose not to do so, the system stalled to an extent; two of the 5 facilities' TB coordinators could not enter new cases on the e-tracker system; inadequate human resources at the hub lab	Poor stability

DOTS: directly observed treatment, short-course

## Discussion

The poor stability identified by this evaluation could mainly be attributed to the inconsistencies between the lab services and the sputum transport system. The challenges at the laboratory were mainly identified as a lack of human resources and a lack of motivation for laboratory staff to work on TB cases. In addition, some of the laboratory staff were reluctant to work on sputum samples because of the COVID-19 pandemic which contributed to a reduction in the efficiency of the TB surveillance system [11].

The inconsistencies of the courier services responsible for sputum transport could be due to miscommunication between the spokes facility TB coordinators and the dispatch rider. The poor acceptability identified in this evaluation could be because most TB training programmes usually focused on the TB coordinators and the team at the DOTS corners, making other health workers feel separated from the system. This could also be because health workers were usually overwhelmed with their primary roles and hence have little to no time to work on TB screening tools and other TB-related activities. An evaluation of the TB surveillance system in Ga West Municipality revealed high acceptability [12].

The system was judged to be flexible because of the level of integration with the HIV surveillance system and the ease with which changes were accepted. The situation was similar when the TB surveillance system of Ga West Municipality was evaluated [12]. An evaluation of the TB surveillance system in South Africa also found the system to be flexible [13].

The poor sensitivity of this system could be explained by the low proportion of all OPD attendants screened for TB. This could be explained by the fact that some of the OPD cases that would have been routinely screened for TB were more likely to be screened for COVID-19 ignoring TB. This could also be due to the low level of knowledge of health workers on how to screen for and diagnose TB. The non-involvement of CBSV in the LANMMA TB surveillance system could contribute to the low sensitivity of the system. Sensitivity was found to be low in other such studies in Ghana [14-17].

This study found that CBSVs were not involved in the LANMMA TB surveillance system. The CBSVs were not prepared to work without any form of incentive. Community-based surveillance volunteers play an intrinsic role in the sensitivity of surveillance systems [18]. When the municipal director of health services was asked about the CBSVs, she stated that the CBSVs had not been involved in the activities of the DHMT due to a lack of funds to motivate them.

**Limitations:** the findings from this study are not generalizable because a single municipality was studied. Also, the findings from this study were validated by only the municipal director of health services and the municipal TB coordinator. Findings from future studies should be validated by all stakeholders involved in the study.

## Conclusion

The TB surveillance system of the LANMMA was observed to have a well-organized structure and line of reporting. Therefore, the system was useful for planning, monitoring, and evaluation of control activities and the development of priorities for TB control programmes. The system was simple and flexible with good data quality. However, stability, sensitivity, predictive value positive, and acceptability were all found to be poor. Even though the downstream flow of information was found to be excellent, the upstream flow of information was found to be poor. Additionally, the system´s performance in terms of achieving its objectives and key indicators, was found to be poor, not meeting the national targets. There is a need to review the sputum transport system and provide more human resources for the hub facility laboratory.

### What is known about this topic

- *It is known that TB surveillance systems provide data that help to assess the performance of TB control interventions;*
- *It is also known that surveillance system evaluations help to measure the performance of surveillance systems to provide data for continuous system improvement*.

### What this study adds

- *This study showed that the shock of COVID-19 and an inefficient sputum transport system negatively impacted the TB surveillance system in a municipality in the Greater Accra Region of Ghana;*
- *The study also found that even though the TB surveillance system of the municipality that was studied did not perform well in some of the system attributes, it was still useful for planning, monitoring, and evaluation of control activities and the development of priorities for TB control programmes*.
